# Ameloblastin induces tumor suppressive phenotype and enhances chemosensitivity to doxorubicin via Src-Stat3 inactivation in osteosarcoma

**DOI:** 10.1038/srep40187

**Published:** 2017-01-05

**Authors:** Toshinori Ando, Yasusei Kudo, Shinji Iizuka, Takaaki Tsunematsu, Hanako Umehara, Madhu Shrestha, Toshihiro Matsuo, Tadahiko Kubo, Shouji Shimose, Koji Arihiro, Ikuko Ogawa, Mitsuo Ochi, Takashi Takata

**Affiliations:** 1 Department of Oral and Maxillofacial Pathobiology, Institute of Biomedical and Health Sciences, Hiroshima University, Hiroshima, Japan; 2 Department of Oral Molecular Pathology, Institute of Health Biosciences, The University of Tokushima Graduate School, Tokushima, Japan; 3 Department of Orthopedic Surgery, Aichi Medical University, Nagakute, Aichi, Japan; 4 Department of Orthopaedic Surgery, Institute of Biomedical and Health Sciences, Hiroshima University, Hiroshima, Japan; 5 Division of Orthopaedic Surgery, National Hospital Organization Kure Medical Center, Kure, Japan; 6 Anatomical Pathology, Hiroshima University Hospital, Hiroshima, Japan; 7 Center of Oral Clinical Examination, Hiroshima University Hospital, Hiroshima, Japan

## Abstract

Ameloblastin (AMBN), the most abundant non-amelogenin enamel matrix protein, plays a role in ameloblast differentiation. Previously, we found that AMBN promoted osteogenic differentiation via the interaction between CD63 and integrin β1, leading to the inactivation of Src; however, how AMBN affects the malignant behavior of osteosarcoma is still unclear. Osteosarcoma affects the bone and is associated with poor prognosis because of the high rate of pulmonary metastases and drug resistance. Here we demonstrated that stable overexpression of AMBN induced apoptosis and suppressed colony formation and cell migration via the inactivation of Src-Stat3 pathway in human osteosarcoma cells. Moreover, AMBN induced chemosensitivity to doxorubicin. Thus, AMBN induced a tumor suppressive phenotype and chemosensitivity to doxorubicin via the AMBN-Src-Stat3 axis in osteosarcoma. Indeed, immunohistochemical expression of AMBN was significantly correlated with better outcome of osteosarcoma patients. Our findings suggest that AMBN can be a new prognostic marker and therapeutic target for osteosarcoma combined with conventional doxorubicin treatment.

Ameloblastin (AMBN), also known as amelin or sheathlin, is the most abundant non-amelogenin enamel matrix protein^1^,^2^,^3^. As shown in AMBN-null mice, AMBN is important for the attachment, polarity, proliferation, and differentiation of ameloblasts^4^. The heparin binding domains of AMBN, in particular, play an essential role in cell attachment^5^. Moreover, AMBN is expressed in osteoblasts during craniofacial bone development^6^ and in mesenchymal cells^7^, suggesting a novel role for AMBN in early bone formation and repair. Furthermore, our previous studies demonstrated that AMBN induces osteogenesis via the AMBN-CD63-integrin β1-src pathway and suppresses Src activity in osteosarcoma cell lines^8^.

Osteosarcoma is a high-grade malignant bone tumor, which predominantly affects adolescents and young adults. Doxorubicin is commonly used as a chemotherapy drug for osteosarcoma, in combination with surgical treatment. However, pulmonary metastasis and recurrence lead to poor prognosis^9^, and the five-year survival rate of patients with osteosarcoma remains around 60–70%^10^. Although our understanding of the molecular basis of osteosarcoma has advanced in recent decades, a breakthrough for treatment has not yet been achieved.

The Src family kinase (SFK) protein, Src kinase, regulates a number of cellular functions, including proliferation, survival, migration and invasion^11^. Because Src is constitutively activated in osteosarcoma, dasatinib, a small-molecule inhibitor of Src kinase activity, suppresses migration and invasion, and induces apoptosis in osteosarcoma cells^12^. Signal transducer and activator of transcription 3 (Stat3) is a transcription factor that regulates proliferation, survival, and angiogenesis^13^,^14^,^15^. Activated Stat3 is also important for cell migration and invasion^16^,^17^, and in S3–54, a small molecule compound targeting the Stat3 DNA-binding domain, suppresses migration and invasion in cancer cells^18^. Stat3 is constitutively activated and essential for the growth and survival of osteosarcoma^19^. Furthermore, Stat3 is activated by Src and contributes to oncogenesis by Src^20^. The Src-Stat3 pathway is persistently activated in osteosarcoma, so inhibitors of both Src and Stat3 induce caspase-3 mediated apoptosis of osteosarcoma cells^21^. Therefore, we hypothesized that AMBN functions as a tumor suppressor in osteosarcoma through the inhibition of Src and its downstream Stat3 activities. This hypothesis is supported by previous findings that (i) tumor formation occurs in AMBN knockout mice^4^, and (ii) AMBN prevents odontogenic tumor development by suppressing cell proliferation and by maintaining cells in a differentiated state through a mechanism that involves Msx2, p21, and p27 (ref. 5). However, the precise function of AMBN in tumors is still unclear. Here, we examined the tumor suppressive role of AMBN in osteosarcoma cells.

## Results

### AMBN induces apoptosis and sensitivity to doxorubicin through the inactivation of Src-Stat3 pathway in osteosarcoma cells

**Figure 1:**
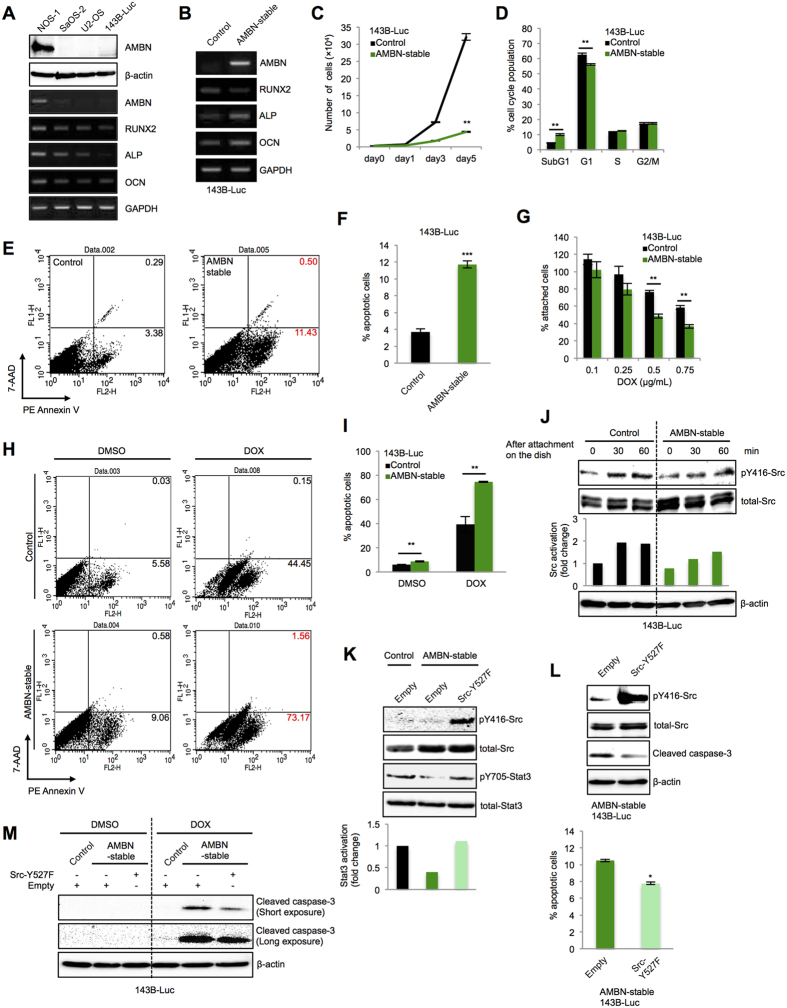
AMBN induces apoptosis and sensitivity to doxorubicin through the Src-Stat3 pathway in osteosarcoma cells. **(A)** The expression of AMBN at the protein level and AMBN, RUNX2, ALP, and OCN at the mRNA level among human osteosarcoma cell lines is shown. **(B)** The expression of AMBN, RUNX2, ALP, and OCN at the mRNA level in control and AMBN-stable 143B-Luc cells was examined. **(C)** Cell growth was counted on days 0, 1, 3, and 5 (*N* = 3). **(D)** Cell cycle distributions were analyzed by PI staining and FACS, and quantified (*N* = 3). **(E)** The apoptotic cell ratio was analyzed by annexin-V/7-AAD staining and FACS, **(F)** and quantified (*N* = 3). **(G)** Control and AMBN-stable 143B-Luc cells were treated with doxorubicin at indicated concentration for 48 hours, then the cells attached on the dishes were counted and quantified (*N* = 3). **(H)** After the treatment with DMSO and doxorubicin (0.5 μg/mL) for 12 h, the apoptotic cell ratio was analyzed by annexin-V/7-AAD staining and FACS, **(I)** and quantified (*N* = 3). **(J)** The expression of pY416-Src and total-Src in control and AMBN-stable 143B-Luc cells after attachment on the culture dish (0, 30, 60 minutes) was examined. Relative expression of pY416-Src (Src activation) is shown. **(K)** Empty vector and Src-Y527F plasmid were transfected into control and AMBN-stable 143B-Luc cells, the expression of pY416-Src, total-Src, pY705-Stat3, total-Stat3 was examined. Relative expression of pY705-Stat3 (Stat3 activation) is shown. **(L)** Empty vector and Src-Y527F plasmid were transfected into AMBN-stable 143B-Luc cells, and the expression of pY416-Src, total-Src, and cleaved caspase-3 was evaluated (upper panel). The apoptotic cell ratio was analyzed by annexin-V/7-AAD staining and FACS, and the representative results are summarized (lower graph) (*N* = 3). **(M)** After the transfection of empty and Src-Y527F into control and AMBN-stable 143B-Luc cells, these cells were treated with DMSO and doxorubicin (0.5 μg/mL) for 24 h. The expression of cleaved caspase-3 was examined. Mean ± SEM (**C,D**,**F**,**G**,**I**,**L**); ***P < 0.001; **P < 0.01; *P < 0.05.

First, AMBN expression among human osteosarcoma cell lines was examined. AMBN expression was high at the mRNA and protein level in NOS-1 cells in association with the expression of osteogenic markers (Runt-related gene 2: RUNX2, Alkaline phosphatase: ALP, Osteocalcin: OCN), while AMBN expression was low in SaOS-2, U2-OS and 143B-Luc cells (Fig. 1A). To elucidate the role of AMBN in osteosarcoma cells, we examined the effects of AMBN overexpression in stably transfected 143B-Luc cells that originally lacked AMBN expression, hereafter referred to as AMBN-stable 143B-Luc cells. Consistent with previous results using SaOS-2 cells^8^, and similar to NOS-1 cells which highly express AMBN, AMBN-stable 143B-Luc cells showed high expression of the osteogenic markers (ALP and OCN) (Fig. 1B). We found that cell growth was markedly suppressed in AMBN-stable 143B-Luc cells compared to control cells (Fig. 1C). We compared the cell cycle distribution of AMBN-stable 143B-Luc cells to that of control cells using FACS analysis. We found that the G1-cell population decreased and the Sub G1-cell population significantly increased in AMBN-stable 143B-Luc cells, and that the apoptotic cell ratio, as detected by Annexin V/7-AAD staining, increased (Figs 1D–F and S1A). Expression of cleaved caspase-3 was strikingly enhanced in AMBN-stable 143B-Luc cells (Fig. S1B). Since AMBN induced apoptosis in osteosarcoma cells, we hypothesized that AMBN-stable 143B-Luc cells would be more sensitive to doxorubicin, which is generally used as a chemotherapy drug for osteosarcoma. As expected, treatment with doxorubicin for 48 h reduced the number of attached AMBN-stable 143B-Luc cells compared to that of control cells, in a dose-dependent manner (Fig. 1G). Furthermore, a higher percentage of the doxorubicin treated, AMBN-stable 143B-Luc cells population (approximately 70%) was apoptotic than the percentage of doxorubicin treated control cell population (approximately 40%) (Fig. 1H,I). Moreover, expression of cleaved caspase-3 was strikingly enhanced by treatment with doxorubicin, but not with DMSO, in AMBN-stable 143B-Luc cells, while expression was only slightly enhanced in control cells treated with doxorubicin (Fig. S1C). Because we previously found that AMBN induces osteogenic differentiation by inactivating Src^8^, we confirmed that Src was inactivated (dephosphorylated at Y416) in AMBN-stable 143B-Luc cells (Fig. 1J). Stat3 is known to affect cell survival and cell migration, and is activated by Src-dependent phosphorylation of tyrosine 705. Likewise, Stat3 activation was disrupted in AMBN-stable 143B-Luc cells and restored by induction of a constitutively active form of Src (Src-Y527F) (Fig. 1K). We also tested whether the overexpression of Src-Y527F rescued the phenotype of AMBN-stable 143B-Luc cells. Ectopic Src-Y527F overexpression in AMBN-stable 143B-Luc cells significantly reduced the apoptotic cell ratio, disrupted expression of cleaved caspase-3 (Fig. 1L). In addition, expression of cleaved caspase-3 in doxorubicin treated AMBN-stable 143B-Luc cells was abrogated by Src-Y527F overexpression (Fig. 1M). Additionally, inducible plasmid was stably transfected into 143B-Luc and U2-OS cells (AMBN-inducible 143B-Luc and U2-OS cells), which induced AMBN expression after Cumate solution treatment. AMBN-inducible 143B-Luc cells showed cleaved caspase-3 expression, suppressed cell growth, induced sensitivity to doxorubicin and inactivated Src-Stat3 pathway (Fig. S2A–E). We previously reported that CD63 was the receptor for AMBN and the AMBN-CD63-Integrin β1-Src axis induced osteogenic differentiation^8^. Therefore, the involvement of CD63 in the AMBN-Src-Stat3 axis was examined. As a result, CD63 was ubiquitously expressed among human osteosarcoma cell lines, and CD63 knockdown reactivated Stat3 in AMBN-inducible 143B-Luc cells (Fig. S3A,B). Thus, AMBN-CD63-Integrinβ1-Src axis continues to Src-Stat3 pathway.

### AMBN regulates apoptosis, sensitivity to doxorubicin, colony formation and cell migration through the inactivation of Src-Stat3 pathway in osteosarcoma cells

**Figure 2:**
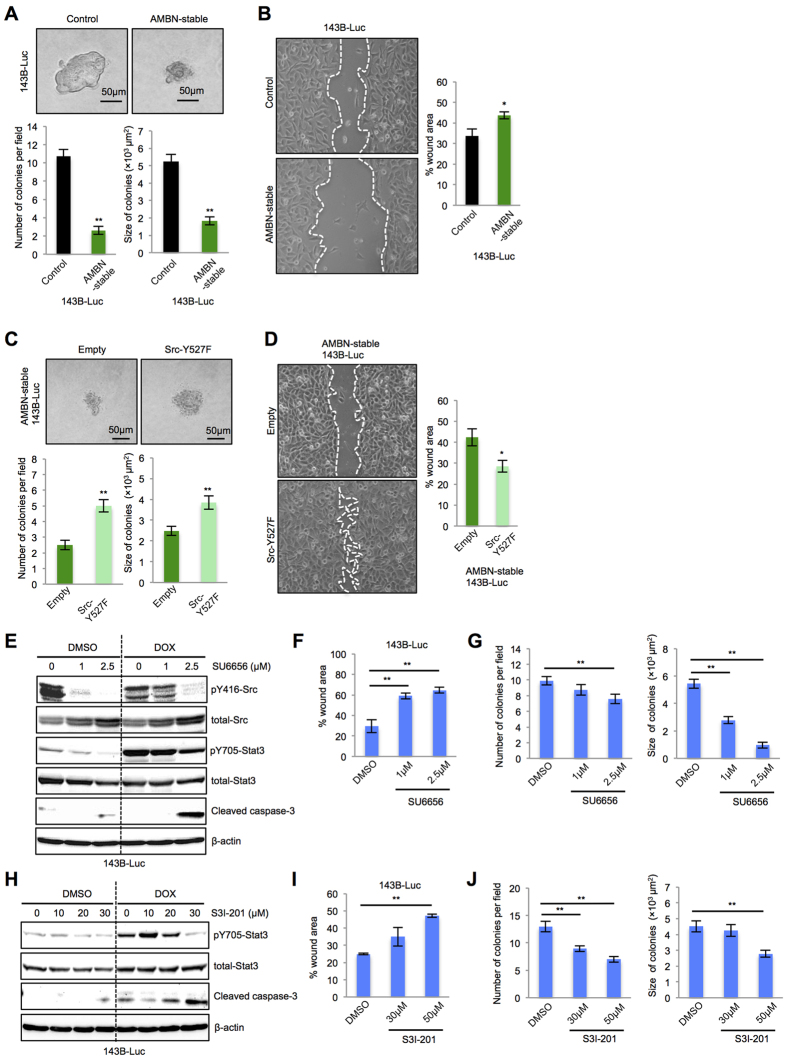
AMBN suppresses colony formation and cell migration through the Src-Stat3 pathway in osteosarcoma cells. **(A)** Colony formation was assessed by soft-agar colony formation assay and representative colonies are shown (upper panels). Quantification of colony number and size is shown (lower graphs) (*N* = 20). **(B)** Cell migration activity was examined by wound healing assay. Representative images at 5 h are shown (left panels) and wound areas were quantified (right graph) (*N* = 3). Original magnification of the left panels: ×100. **(C)** Empty vector and Src-Y527F plasmid were transfected into AMBN-stable 143B-Luc cells, colony formation was assessed and representative colonies are shown (upper panels). Quantification of colony number and size is shown (lower graphs) (*N* = 20). **(D)** Cell migration activity was examined. Representative images at 5 h are shown (left panels) and wound areas were quantified (right graph) (*N* = 3). Original magnification of the left panels: ×100. **(E)** 143B-Luc cells were pretreated with SU6656 at indicated concentration for 24 h. The pretreated 143B-Luc cells were subsequently treated with DMSO or doxorubicin (0.5 μg/mL) for 24 h and the expression of pY416-Src, total-Src, pY705-Stat3, total-Stat3, and cleaved caspase-3 was examined. **(F)** Cell migration activity of pretreated 143B-Luc cells was examined. Wound areas at 5 h were quantified in the graphs (*N* = 3). **(G)** Colony formation in pretreated 143B-Luc cells was analyzed and representative colonies were shown (upper panels). Quantification of colony number and size was shown (lower panels) (*N* = 20). **(H)** 143B-Luc cells were pretreated with S3I-201 at indicated concentration for 24 h. The pretreated 143B-Luc cells were subsequently treated with doxorubicin (0.5 μg/mL) for 24 h and the expression of pY705-Stat3, total-Stat3 and cleaved caspase-3 was examined. **(I)** Cell migration activity of pretreated 143B-Luc cells was examined. Wound areas at 5 h were quantified in the graphs (*N* = 3). **(J)** Colony formation in pretreated 143B-Luc cells was analyzed and representative colonies were shown (upper panels). Quantification of colony number and size was shown (lower panels) (*N* = 20). Mean ± SEM (**A–D,F,G,I**,**J**); **P < 0.01; *P < 0.05.

More phenotypes in AMBN-stable and AMBN-inducible 143B-Luc cells were examined. Colony formation, determined by colony number and size in soft-agar colony formation assays, and cell migration, determined by wound healing assays, were inhibited in AMBN-stable and AMBN-inducible 143B-Luc cells (Figs 2A,B and S4). Also, ectopic Src-Y527F overexpression in AMBN-stable 143B-Luc cells enhanced colony formation (Fig. 2C) and cell migration (Fig. 2D). Moreover, the treatment of control 143B-Luc cells with the Src inhibitor, SU6656, recapitulated the phenotype of AMBN-stable 143B-Luc cells. In particular, treatment of 143B-Luc cells with this inhibitor suppressed Src and Stat3 activation and induced the expression of cleaved caspase-3, and sensitivity to doxorubicin with markedly enhanced expression of cleaved caspase-3 (Fig. 2E). SU6656 also suppressed both cell migration (Fig. 2F) and colony formation (Figs 2G and S5A). The treatment of control 143B-Luc cells with Stat3 inhibitor, S3I-201, also induced apoptosis, sensitivity to doxorubicin, suppressed cell migration and colony formation (Figs 2H–J and S5B). Taken together, these results suggest that AMBN induces caspase-3 mediated apoptosis, sensitivity to doxorubicin, and suppresses colony formation and cell migration through Src-Stat3 inactivation.

### AMBN Knockdown rescues from apoptosis, promotes colony formation and cell migration through the activation of Src-Stat3 pathway in NOS-1 cells

**Figure 3:**
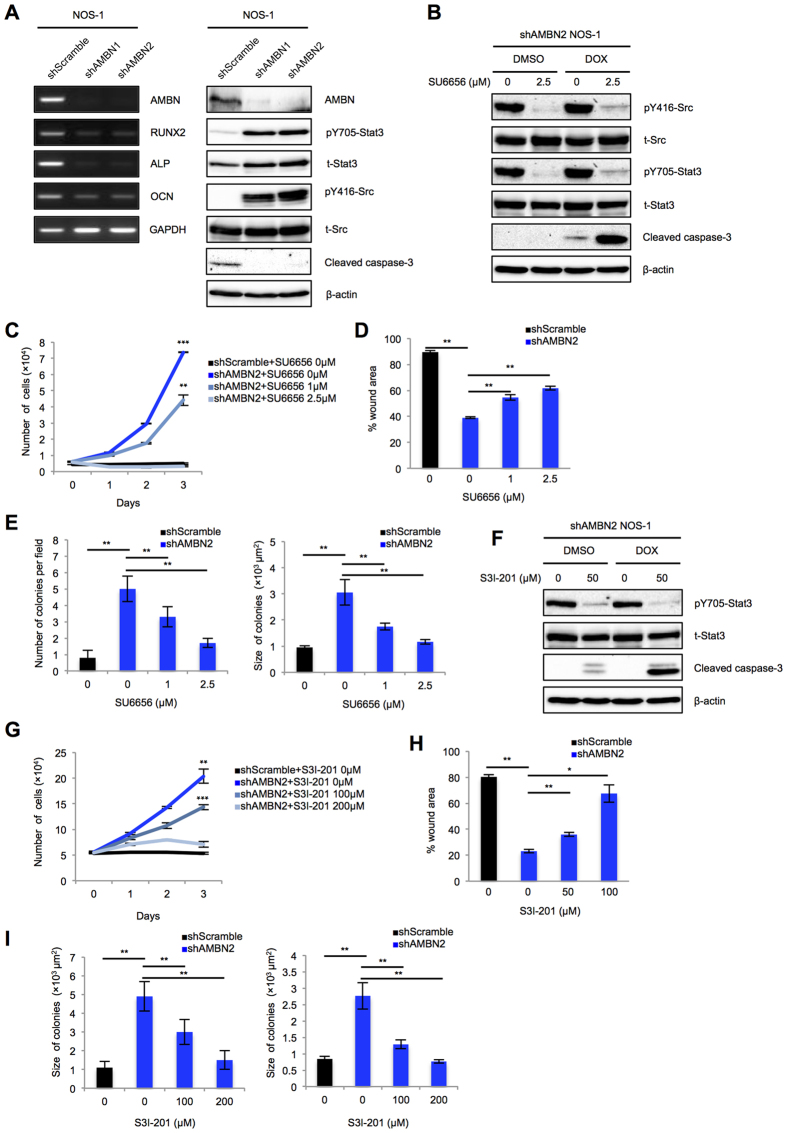
AMBN knockdown suppresses apoptosis, sensitivity to doxorubicin, and promotes cell migration and colony formation through the Src-Stat3 pathway in NOS-1 cells. (**A**) The expression of AMBN, RUNX2, ALP, and OCN at the mRNA level (left panel) and AMBN, pY705-Stat3, total-Stat3, pY416-Src, total-Src and cleaved caspase-3 at the protein level in shscramble, shAMBN1, and shAMBN2 NOS-1 cells was examined (right panel). (**B**,**F**) shAMBN2 NOS-1 cells were pretreated with SU6656 or S3I-201 at indicated concentration for 24 h. The pretreated shAMBN-2 NOS-1 cells were subsequently treated with DMSO or doxorubicin (0.5 μg/mL) for 24 h and the expression of pY416-Src, total-Src, pY705-Stat3, total-Stat3, and cleaved caspase-3 was examined. (**C**,**G**) shscramble and shAMBN-2 NOS-1 cells were treated with SU6656 o S3I-201 at indicated concentration and cell growth was counted on days 0, 1, 2, and 3 (*N* = 3). (**D**,**H**) Cell migration activity of pretreated shscramble and shAMBN-2 NOS-1 cells was examined. Wound areas at 5 h were quantified in the graphs (*N* = 3). (**E**,**I**) Colony formation in pretreated shscramble and shAMBN-2 NOS-1 cells was analyzed. Quantification of colony number (left panel) and size (right panel) was shown (*N* = 20). Mean ± SEM (**C–E**,**G–I**); **P < 0.01; *P < 0.05.

Stable knockdown of AMBN in NOS-1 cells was performed using shRNA transfection, which reduced the expression of AMBN at the mRNA and protein levels in association with the reduction of cleaved caspase-3, the activation of Src and Stat3, and the suppression of osteogenic markers (RUNX2, ALP and OCN) (Fig. 3A). Treatment of shAMBN2 NOS-1 cells with SU6656 suppressed Src and Stat3 activation and induced sensitivity to doxorubicin with markedly enhanced expression of cleaved caspase-3 (Fig. 3B). Also, stable knockdown of AMBN in NOS-1 cells significantly promoted cell proliferation, migration and colony formation, and treatment of shAMBN2 NOS-1 cells with SU6656 suppressed these phenotypes in dose-dependent manner (Figs 3C–E and S6A,B). Moreover, treatment of shAMBN2 NOS-1 cells with S3I-201 suppressed Stat3 activation, induced sensitivity to doxorubicin with significantly enhanced expression of cleaved caspase-3 and inhibited cell proliferation, migration and colony formation (Figs 3F–I and S6C,D).

### AMBN suppresses tumor growth and pulmonary metastases of osteosarcoma cells *in vivo*, and AMBN expression correlates with pulmonary metastases in clinical osteosarcoma cases

**Figure 4:**
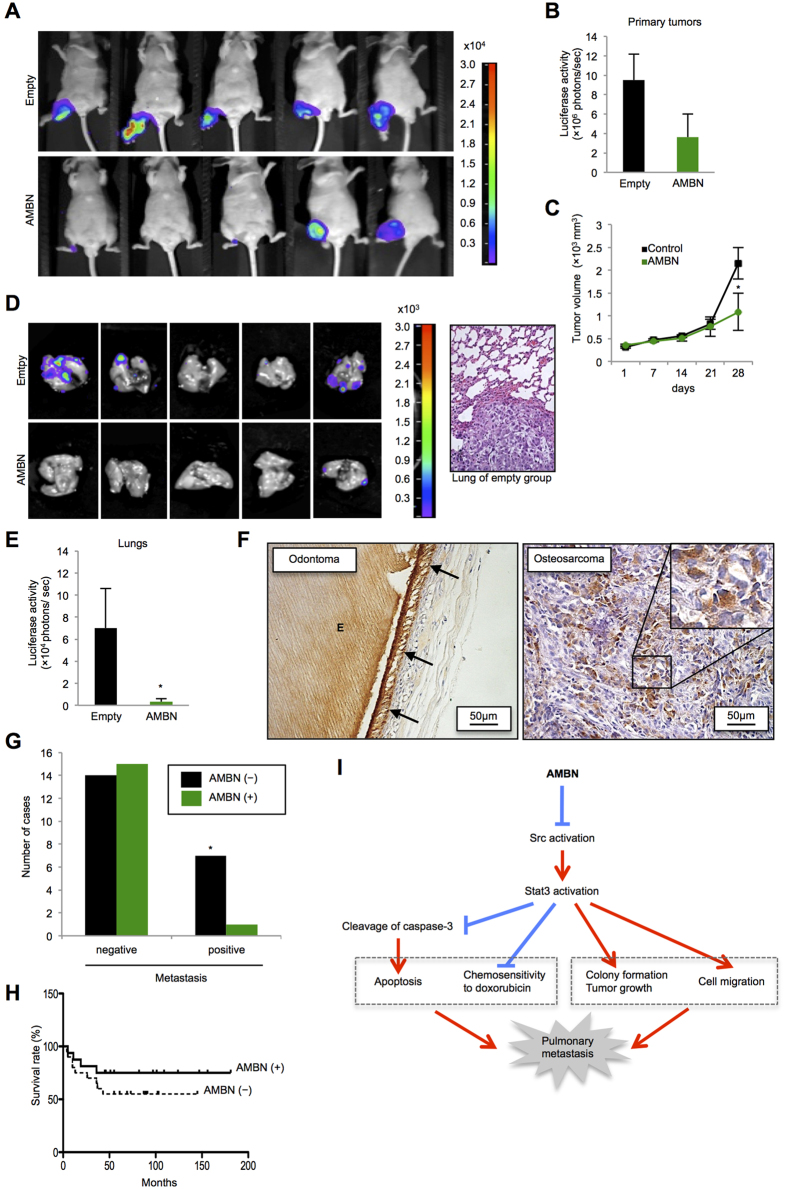
AMBN inhibits tumor growth and pulmonary metastases *in vivo*, and AMBN expression is correlated with pulmonary metastasis and prognosis in human osteosarcoma cases. (**A**) Photons from luciferase in primary tumors of empty and AMBN groups after 28 days were captured. (**B**) Quantification of photons from luciferase in the primary tumors is shown (*N* = 5). (**C**) The volume of primary tumors at indicated days is shown (*N* = 5). (**D**) Photons from luciferase in the lungs after 28 days were captured (left panels) and representative metastatic focus of lung in empty group is shown (right panel). Original magnification of the right panel: ×200. (**E**) Quantification of photons from luciferase in the lungs is shown (*N* = 5). (**F**) AMBN expression in 37 osteosarcoma cases was evaluated by immunohistochemical analysis. Specificity of anti-AMBN antibody was confirmed by using odontoma. AMBN protein was stained in ameloblasts and enamel. Arrow indicates ameloblasts and “E” indicates enamel (left panel). Representative tissue of AMBN staining in osteosarcoma cases is shown. AMBN was expressed in cytoplasm of osteosarcoma cells (right panel). (**G**) The correlation between AMBN expression and pulmonary metastasis is shown (*N* = 37, chi-squared test). *P < 0.05. (**H**) The correlation between AMBN expression and survival rate for 37 patients with osteosarcoma was analyzed and shown as a Kaplan-Meier graph (*N* = 37, log-rank test). (**I**) In this model, AMBN inhibits Src activation (dephosphorylation at Y416) followed by Stat3 inactivation (dephosphorylation at Y705). Continuous inactivation of the Src-Stat3 pathway induces caspase-3 mediated apoptosis and chemosensitivity to doxorubicin, while suppresses colony formation, tumor growth and cell migration. These activities lead to the reduction of pulmonary metastasis. Mean ± SEM (**B**,**C**,**E**); *P < 0.05.

We used 143B-Luc cells for *in vivo* experiments because these cells originally were generated by transformation of HOS cells, one of the human osteosarcoma cell lines, via the retroviral oncogene v-Ki-ras, and because these cells have high tumorigenicity and metastatic potential to lung^22^. The AMBN-pcDNA3.1 plasmid or the empty vector was transiently transfected into 143B-Luc cells. Then, approximately 1.5 × 10^6^ cells were injected into the right hind leg of five-week-old male athymic nude mice at day 0. After 28 days, a luciferase signal was detected in primary tumors and pulmonary metastases (Fig. 4A). The tumors of the AMBN-overexpressing group showed slightly lower luciferase activity in the primary tumor site (no significant difference) and significantly lower tumor volume than that of control group (Fig. 4B,C). Interestingly, the tumors of the control group, but not of the AMBN-overexpressing group, destroyed the leg bone (Fig. S7A). All tumors of the control group metastasized to the lung, whereas only one tumor of the AMBN-overexpressing group metastasized to the lung (Fig. 4D). Hematoxylin and eosin (HE) staining of pulmonary metastasis from the control group is shown in Fig. 4D. The average luciferase activity in pulmonary metastases of the AMBN-overexpressing tumors was markedly lower than that of control tumors (Fig. 4E).

We also injected approximately 1.5 × 10^6^ cells of the control cells and the AMBN-stable 143B-Luc cells into the right hind knee of five-week-old male athymic nude mice, and all mice were sacrificed at day 23. AMBN mRNA expression in tumors was confirmed by RT-PCR (Fig. S7B). The primary volume and weight of tumors collected from the AMBN-stable group were significantly reduced compared to those of tumors from the control group (Fig. S7B–D). The number of metastatic foci in the lung was also significantly reduced in the AMBN-stable group (Fig. S7E). Relatively more necrotic area was observed in the primary tumor in AMBN-stable group (Fig. S7F). These results suggest that ectopic overexpression of AMBN inhibits primary tumor growth and pulmonary metastases of osteosarcoma *in vivo*.

Finally, We assessed AMBN expression in human primary osteosarcoma cases and its correlation with lung metastasis and prognosis. Thirty-seven biopsy samples of primary osteosarcoma were used (Table S1). We checked the specificity of an AMBN antibody in ameloblasts of odontoma. The antibody specifically recognized AMBN in ameloblasts (indicated by arrows) and AMBN that was secreted into enamel (indicated by “E”) (Fig. 4F). Using this antibody, we examined AMBN expression in primary osteosarcoma cases using immunohistochemical analysis. AMBN was clearly detected in the cytoplasm of osteosarcoma cells. Positive AMBN expression was observed in 16 out of 37 osteosarcoma cases (Fig. 4F and Table 1). Pulmonary metastasis was observed in 1 out of 16 (6.3%) cases with positive AMBN expression, and in 7 out of 21 (33.3%) cases that lacked AMBN expression (Fig. 4G and Table 1). Interestingly, osteosarcoma cases with positive AMBN expression showed better prognosis than those without (Fig. 4H), but the difference in prognosis was not statistically significant as determined by Log-rank test.

## Discussion

AMBN is a cell adhesion molecule that suppresses proliferation and maintains the differentiated phenotype of ameloblasts^4^,^5^. Fisher *et al*. proposed the grouping of calcium-binding phosphoproteins of teeth and bone into the small integrin-binding ligand, N-linked glycoproteins (SIBLINGs) family, including bone sialoprotein/BSP, osteopontin/OPN, dentin matrix protein 1/DMP1, dentin sialophosphoprotein/DSPP, matrix extracellular phosphoglycoprotein/MEPE, and enamelin/ENAM^23^,^24^. Moreover, Kawasaki and Weiss proposed the grouping of the secretory calcium-binding phosphoprotein (SCPP) gene family, including the dentin/bone or acidic SCPPs and the milk/saliva/enamel SCPPs^25^,^26^. SCPPs are all secretory proteins and bind to calcium ions with their phospho-Ser residues. All of the human SCPP genes, except for Amelogenin, are clustered on the long arm of human chromosome 4, and AMBN is also located on human chromosome 4 as a member of milk/saliva/enamel SCPPs, next to the dentin/bone or acidic SCPPs (SIBLINGs)^25^,^26^,^27^. Therefore, AMBN was expected to be involved in osteogenesis. Using osteosarcoma cell lines, we previously found that AMBN induces osteogenic differentiation through the AMBN-CD63-Integrin β1-src axis^8^. Additionally, structural calculations of human and mouse AMBN allowed identified the CD63-interaction domain^28^.

Here, we have demonstrated that AMBN induced apoptosis and doxorubicin sensitivity, and suppressed colony formation and cell migration in osteosarcoma cells via inactivation of the Src-Stat3 pathway. Stat3 is activated by Src-mediated phosphorylation at Y705. Activated Stat3 forms homodimers and is transferred into the nucleus, where it promotes the transcription of target genes required for cell growth, survival, and migration^13^,^14^,^15^,^16^,^17^,^20^. Src and Stat3 are constitutively activated and have oncogenic roles in human osteosarcoma^21^. In this study, the activation of Src-Stat3 signaling was observed among all tested human osteosarcoma cell lines, except NOS-1 cells that highly express AMBN (Fig. 1A). Furthermore, Stat3 is constitutively activated in osteosarcoma, rhabdomyosarcoma, and other soft-tissue sarcomas, and the Stat3 inactivation pathway results in apoptosis through the cleavage of caspase-3^19^. In a separate study, IL-6-dependent Stat3 activation in mesenchymal stem cell-derived osteosarcoma cells protects tumor cells from drug-induced apoptosis, and promoted proliferation and metastasis^29^. In this study, we found that inactivation of the Src-Stat3 pathway by AMBN overexpression induced caspase-3 mediated apoptosis and doxorubicin sensitivity, and suppressed colony formation and cell migration. In a previous study, AMBN inhibited proliferation of odontogenic tumor cells through the up-regulation of p21 and p27^5^; therefore, we also analyzed the relation between AMBN and p21 in osteosarcoma. In contrast to previous reports, we found that AMBN-stable 143B-Luc cells did not arrest at the G1 phase of the cell cycle (Fig. 1D). Up-regulation of the Cdk inhibitors, p21 and p27, inhibits G1 to S phase progression of the cell cycle via hypophosphorylation of pRb. Moreover, mutations in *RB1* (70–90% of cases), as well as in *TP53* (50–70% of cases), are highly associated in human osteosarcoma^30^,^31^,^32^, and SaOS-2 cells also lack functional pRb expression^33^. Thus, the importance of p21 and p27 as tumor suppressors in osteosarcoma is still unclear^34^ and AMBN is thought to act independent of p21 and p27 in osteosarcoma.

We previously demonstrated that binding of AMBN to CD63-Integrin β1 induces the inactivation of Src^8^, suggesting that AMBN induces inactivation of the Src-Stat3 pathway via the AMBN-CD63-Integrin β1-Src axis. Importantly, in this study we demonstrated that in cases of osteosarcoma with positive AMBN expression, there are lower frequency of pulmonary metastases and better prognosis (Fig. 4G,H and Table 1). Pulmonary metastasis and osteosarcoma recurrence remain the major causes of mortality in osteosarcoma. Therefore, we suggest that the inactivation of the Src-Stat3 pathway by AMBN, combined with conventional doxorubicin treatment, can be a therapeutic modality for osteosarcoma. AMBN is a secreted protein, which may be an advantage for clinical use.

While a well-differentiated osteosarcoma generally is classified as a low-grade tumor, dedifferentiated tumors usually fall into the high-grade category^35^. Therefore, loss of differentiation has a powerful influence on the prognosis of osteosarcoma. Moreover, recent investigations have focused on the therapeutic potential of differentiation inducing molecules, because lack of terminal differentiation may be responsible for osteosarcoma tumorigenesis^36^. Since AMBN induces osteogenic differentiation of osteosarcoma cells^8^, it is possible that AMBN acts antagonistically to the oncogenic process through the induction of osteogenic differentiation, and through the suppression of survival and migration.

Mutant mice expressing a truncated form of AMBN lacking portions of the protein encoded by exons 5 and 6 develop soft tissue tumors in the buccal vestibule of the maxilla with age^4^, and AMBN plays an important role in preventing odontogenic tumor development by suppressing cell proliferation^5^. Therefore, the complete loss of AMBN in the bone tissue, which originally expresses AMBN, may induce osteosarcoma development. It would be intriguing to examine AMBN full-knockout mice.

In this study, we determined the underlying molecular mechanisms of a novel function for AMBN in osteosarcoma; stable overexpression of AMBN inactivated the Src-Stat3 axis and subsequently induced caspase-3 mediated apoptosis and sensitivity to doxorubicin, while inhibiting colony formation, cell migration, tumor growth and pulmonary metastases. These activities led to better prognosis for osteosarcoma and to the reduction of pulmonary metastasis (Fig. 4I). Osteosarcoma with pulmonary metastases still shows a poor prognosis; therefore, AMBN can be a useful prognostic marker and a target for the treatment of the patients with osteosarcoma when combined with conventional doxorubicin treatment.

## Materials and Methods

### Reagents and antibodies

An expression vector encoding the constitutively active, mutant form of Src (Src-Y527F) was donated by Addgene, Inc. (Cambridge, MA). Commercial antibodies were purchased from the following suppliers: anti-AMBN polyclonal antibody and anti-CD63 (MX-49.129.5) monoclonal antibody from Santa Cruz Biotechnology (Dallas, TX); anti-β-actin monoclonal antibody and anti-FLAG monoclonal antibody (M2) from Sigma (St. Louis, MO); and anti-cleaved caspase-3 polyclonal antibody, anti-Src polyclonal antibody, polyclonal antibody specific to phospho-Tyr416 Src, anti-Stat3 polyclonal antibody, and polyclonal antibody specific to phospho-Tyr705 Stat3 from Cell Signaling Technology (Danvers, MA). Commercial reagents were purchased from the following suppliers: doxorubicin from LC laboratories (Woburn, MA); Z-DEVD-FMK from Medical and Biological Laboratories Co., LTD. (Aichi, Japan). SU6656 and S3I-201 were purchased from Sigma (St. Louis, MO).

### Cell lines

Human osteosarcoma cell lines (NOS-1, SaOS-2, U2-OS) were provided by Cell Bank, RIKEN BioResource Center (Ibaraki, Japan). For *in vitro* and *in vivo* experiments, a human osteosarcoma cell line, 143B-Luc, which is a luciferase-expression clone of 143B^22^, was provided by Dr. Takahiro Ochiya (National Cancer Center Research Institute Tokyo, Japan). They were cultured in the medium described previously^8^.

### Cell growth

Cells (5.0 × 10^3^) were plated onto a 24-well multi-well plate (Falcon, Franklin Lakes, NJ) and allowed to attach for 24 h. The culture medium was replaced with fresh medium, and the trypsinized cells were counted using a Coulter Z1 Cell Counter (Beckman Coulter, Inc., Brea, CA) at days 0, 1, 3, and 5.

### Flow cytometric analysis

Flow cytometric analysis was carried out as described previously^37^.

### Annexin V and 7-AAD dual-staining assay

Annexin V and 7-AAD dual-staining assay was performed as described previously^37^.

### Soft-agar colony formation assay

Cells (2.0 × 10^4^) were mixed with Dulbecco’s modified eagle medium (DMEM) (Nissui, Tokyo, Japan) containing 0.36% agar, plated into 6-well multi-well plate, and cultured for 7days. The colonies larger than 25 μm in diameter were counted.

### RT-PCR

RT-PCR was carried out as described previously^8^. The primer sequences for human AMBN, RUNX2, ALP, OCN, and GAPDH were described previously^8^.

### Western blot analysis

Western blotting was carried out as described previously^8^. Twenty micrograms of protein was subjected to 10% polyacrylamide gel electrophoresis and then electroblotted onto a nitrocellulose filter. For detection of the immunocomplex, the ECL western blotting detection system (GE Healthcare, Tokyo, Japan) was used.

### Transfection and selection

Cell transfection was performed using Lipofectamine 3000 Reagent (Thermo Fisher Scientific, Waltham, MA) according to the manufacture’s instruction. AMBN-pcDNA3.1 plasmid into 143B-Luc cells, and the cells were treated with Zeocin (16.7 μg/mL; Invitrogen) for 2–3 weeks and some clonal colonies were selected and isolated, then stably AMBN overexpressing 143B-Luc cells were established. Also, AMBN was subcloned into a inducible vector, PBQM812A-1 (System Biosciences, LLC, Palo Alto, CA). And AMBN-inducible plasmid was transfected into 143B-Luc cells, and the cells were treated with puromycin (0.5 μM, Wako Pure Chemical Industries, Ltd., Osaka, Japan) for 2–3 weeks and some clonal colonies were selected and isolated, then stably AMBN-inducible 143B-Luc cells were established. The cells were treated with Cumate solution (150–300 μg/mL, PBQM100A-1, System Biosciences, LLC, Palo Alto, CA) for 3 days, then AMBN expression was confirmed by western blot analysis.

### Silencing by shRNA

PBSI505A-1 vector (System Biosciences, LLC, Palo Alto, CA) was used for knockdown experiment. shRNA target sequences used for this study are as follows: shscramble, 5′-GTTCTCCGAACGTGTCACGT-3′, shAMBN1, 5′-CCAAATAGCCCGTTTGATTTC-3′, shAMBN2, 5′-CTGAATTAGCTGATGTTTATA-3′, shCD63, 5′-GCTATGTGTTTAGAGATAAGG-3′. The plasmids were transfected into NOS-1 cells and cultured with puromycin (0.5 μM) for 2–3 weeks, then some colonies were isolated and cultured, finally shscramble, shAMBN1 and shAMBN2 NOS-1 cells were established. Knockdown efficacy was confirmed by RT-PCR and Western blot analysis.

### Wound healing assay

For the wound healing experiment, cells were seeded on 6-well plates and allowed to grow to complete confluence. Subsequently, a plastic pipette tip was used to scratch the cell monolayer to create a cleared area, and the wounded cell layer was washed with fresh media to remove loose cells. Immediately after the wounding (0 h) and after incubation of the cells at 37 °C for 5 h, phase-contrast images (10 × field) of the wound healing process were digitally acquired with an inverted microscope. The wound area was measured by Image J software, set at 100% for 0 h, and the mean percentage of the total area was calculated.

### Tissue samples

Osteosarcoma tissue samples were retrieved from the Surgical Pathology Registry of Hiroshima University Hospital. The informed consent was obtained from all subjects and all experimental protocols were approved by the Ethical Committee of Hiroshima University Hospital (registration number: E-8). All methods were carried out in accordance with the approved guidelines. Clinical data of 37 osteosarcoma cases, provided by the Orthopaedic Surgery Department of Hiroshima University Hospital, were used for the immunohistochemical analysis.

### Immunohistochemical staining

Immunohistochemical detection of AMBN in the tissue samples was performed on 4.5-μm sections mounted on silicone-coated glass slides using a streptavidin-biotin peroxidase technique, as described previously^8^. For the immunohistochemical study, anti-AMBN polyclonal antibody was used.

### Animal model

Animal use and procedures were approved by Hiroshima University Research Facilities of Laboratory Animal Science. All methods were performed in accordance with the approved guidelines. Five-week-old male athymic nude mice (CLEA Japan, Shizuoka, Japan) were used. Under intraperitoneal anesthesia with 10% pentobarbital sodium (Somnopentyl^®^: 0.6 mL/kg, Schering-Plough Animal Health, USA), the anesthetized animals were injected with 1.5 × 10^6^ cells into the right hind leg on day 0. For *in vivo* imaging, the mice were intraperitoneally injected with D-luciferin (150 mg/kg; Promega). Ten minutes later, photon levels from luciferase was counted using NightOWL II LB 983 (Berthold technologies) according to the manufacturer’s instructions. The data were analyzed using IndiGO2 (Berthold Technology). Bone erosion caused by the primary tumor was visualized using inspeXio SMX-90CT (Shimadzu). At the end of the experiment, the primary tumors and lungs were analyzed *in vivo* by bioluminescent imaging and also checked histologically.

### Statistical analyses

Data are presented as mean values ± SEM. Statistical analyses were conducted using Student *t* test, chi-squared test, or log-rank test. *P* < 0.05 was considered significant.

## Additional Information

**How to cite this article**: Ando, T. *et al*. Ameloblastin induces tumor suppressive phenotype and enhances chemosensitivity to doxorubicin via Src-Stat3 inactivation in osteosarcoma. *Sci. Rep.* **7**, 40187; doi: 10.1038/srep40187 (2017).

**Publisher's note:** Springer Nature remains neutral with regard to jurisdictional claims in published maps and institutional affiliations.

## Supplementary Material

Supplementary Information

## Figures and Tables

**Table 1:** Correlation between Ameloblastin expression and lung metastasis.

	Total	Ameloblastin expression		*P* value
		−	+	
Osteosarcoma	37	21	16	
Lung metastasis				
Negative	29	14	15	0.047
Positive	8	7	1	

## References

[b1] Cerný R., Slaby I., Hammarström L. & Wurtz T. A novel gene expressed in rat ameloblasts codes for proteins with cell binding domains. J Bone Miner Res 11, 883–91 (1996). 8797107 10.1002/jbmr.5650110703

[b2] Fong C. D., Slaby I. & Hammarström L. Amelin: an enamel-related protein, transcribed in the cells of epithelial root sheath. J Bone Miner Res 11, 892–8 (1996). 8797108 10.1002/jbmr.5650110704

[b3] Krebsbach P. H. . Full-length sequence, localization, and chromosomal mapping of ameloblastin. A novel tooth-specific gene. J Biol Chem 271, 4431–5 (1996). 8626794 10.1074/jbc.271.8.4431

[b4] Fukumoto S. . Ameloblastin is a cell adhesion molecule required for maintaining the differentiation state of ameloblasts. J Cell Biol 167, 973–83 (2004). 15583034 10.1083/jcb.200409077 PMC2172447

[b5] Sonoda A. . Critical role of heparin binding domains of ameloblastin for dental epithelium cell adhesion and ameloblastoma proliferation. J Biol Chem 284, 27176–84 (2009). 19648121 10.1074/jbc.M109.033464 PMC2785645

[b6] Spahr A., Lyngstadaas S. P., Slaby I. & Pezeshki G. Ameloblastin expression during craniofacial bone formation in rats. Eur J Oral Sci 114, 504–11 (2006). 17184233 10.1111/j.1600-0722.2006.00403.x

[b7] Tamburstuen M. V. . Ameloblastin expression and putative autoregulation in mesenchymal cells suggest a role in early bone formation and repair. Bone 48, 406–13 (2011). 20854943 10.1016/j.bone.2010.09.007 PMC4469498

[b8] Iizuka S. . Ameloblastin regulates osteogenic differentiation by inhibiting Src kinase via cross talk between integrin beta1 and CD63. Mol Cell Biol 31, 783–92 (2001). 10.1128/MCB.00912-10 PMC3028634 21149578

[b9] Lewis V. O. What’s new in musculoskeletal oncology. J Bone Joint Surg Am 91, 1546–56 (2009). 19487537 10.2106/JBJS.I.00375

[b10] Bielack S. S. . Prognostic factors in high-grade osteosarcoma of the extremities or trunk: an analysis of 1,702 patients treated on neoadjuvant cooperative osteosarcoma study group protocols. J Clin Oncol 20, 776–90 (2002). 11821461 10.1200/JCO.2002.20.3.776

[b11] Thomas S. M. & Brugge J. S. Cellular functions regulated by Src family kinases. Annu Rev Cell Dev Biol 13, 513–609 (1997). 9442882 10.1146/annurev.cellbio.13.1.513

[b12] Shor A. C. . Dasatinib inhibits migration and invasion in diverse human sarcoma cell lines and induces apoptosis in bone sarcoma cells dependent on SRC kinase for survival. Cancer Res 67, 2800–8 (2007). 17363602 10.1158/0008-5472.CAN-06-3469

[b13] Darnell J. E. Jr. STATs and gene regulation. Science 277, 1630–5 (1997). 9287210 10.1126/science.277.5332.1630

[b14] Darnell J. E. Jr. Transcription factors as targets for cancer therapy. Nat Rev Cancer 2, 740–9 (2002). 12360277 10.1038/nrc906

[b15] Bromberg J. & Darnell J. E. Jr. The role of STATs in transcriptional control and their impact on cellular function. Oncogene 19, 2468–73 (2000). 10851045 10.1038/sj.onc.1203476

[b16] Cheng G. Z. . Twist is transcriptionally induced by activation of STAT3 and mediates STAT3 oncogenic function. J Biol Chem 283, 14665–73 (2008). 18353781 10.1074/jbc.M707429200 PMC2386910

[b17] Xie T. X. . Stat3 activation regulates the expression of matrix metalloproteinase-2 and tumor invasion and metastasis. Oncogene 23, 3550–60 (2004). 15116091 10.1038/sj.onc.1207383

[b18] Huang W. . A small molecule compound targeting STAT3 DNA-binding domain inhibits cancer cell proliferation, migration, and invasion. ACS Chem Biol 9, 1188–96 (2014). 24661007 10.1021/cb500071v PMC4033648

[b19] Chen C. L. . Signal transducer and activator of transcription 3 is involved in cell growth and survival of human rhabdomyosarcoma and osteosarcoma cells. BMC Cancer 7, 111 (2007). 17598902 10.1186/1471-2407-7-111 PMC1964761

[b20] Yu C. L. . Enhanced DNA-binding activity of a Stat3-related protein in cells transformed by the Src oncoprotein. Science 269, 81–3 (1995). 7541555 10.1126/science.7541555

[b21] Fossey S. L. . Characterization of STAT3 activation and expression in canine and human osteosarcoma. BMC Cancer 9, 81 (2009). 19284568 10.1186/1471-2407-9-81 PMC2666757

[b22] Osaki M. . MicroRNA-143 regulates human osteosarcoma metastasis by regulating matrix metalloprotease-13 expression. Mol Ther 19, 1123–30 (2011). 21427707 10.1038/mt.2011.53 PMC3129798

[b23] Fisher L. W., Torchia D. A., Fohr B., Young M. F. & Fedarko N. S. Flexible structures of SIBLING proteins, bone sialoprotein, and osteopontin. Biochem Biophys Res Commun 280, 460–5 (2001). 11162539 10.1006/bbrc.2000.4146

[b24] Fisher L. W. & Fedarko N. S. Six genes expressed in bones and teeth encode the current members of the SIBLING family of proteins. Connect Tissue Res 44 Suppl 1, 33–40 (2003). 12952171

[b25] Kawasaki K. & Weiss K. M. Mineralized tissue and vertebrate evolution: the secretory calcium-binding phosphoprotein gene cluster. Proc Natl Acad Sci USA 100, 4060–5 (2003). 12646701 10.1073/pnas.0638023100 PMC153048

[b26] Kawasaki K. & Weiss K. M. SCPP gene evolution and the dental mineralization continuum. J Dent Res 87, 520–31 (2008). 18502959 10.1177/154405910808700608

[b27] Huq N. L., Cross K. J., Ung M. & Reynolds E. C. A review of protein structure and gene organisation for proteins associated with mineralised tissue and calcium phosphate stabilisation encoded on human chromosome 4. Arch Oral Biol 50, 599–609 (2005). 15892946 10.1016/j.archoralbio.2004.12.009

[b28] Zhang X., Diekwisch T. G. & Luan X. Structure and function of ameloblastin as an extracellular matrix protein: adhesion, calcium binding, and CD63 interaction in human and mouse. Eur J Oral Sci 119 Suppl 1, 270–9 (2011). 22243256 10.1111/j.1600-0722.2011.00889.x PMC3402545

[b29] Tu B., Du L., Fan Q. M., Tang Z. & Tang T. T. STAT3 activation by IL-6 from mesenchymal stem cells promotes the proliferation and metastasis of osteosarcoma. Cancer Lett 325, 80–8 (2012). 22743617 10.1016/j.canlet.2012.06.006

[b30] Clark J. C., Dass C. R. & Choong P. F. A review of clinical and molecular prognostic factors in osteosarcoma. J Cancer Res Clin Oncol 134, 281–97 (2008). 17965883 10.1007/s00432-007-0330-x PMC12161636

[b31] Kansara M. & Thomas D. M. Molecular pathogenesis of osteosarcoma. DNA Cell Biol 26, 1–18 (2007). 17263592 10.1089/dna.2006.0505

[b32] Calo E. . Rb regulates fate choice and lineage commitment *in vivo*. Nature 466, 1110–4 (2010). 20686481 10.1038/nature09264 PMC2933655

[b33] Shew J. Y. . C-terminal truncation of the retinoblastoma gene product leads to functional inactivation. Proc Natl Acad Sci USA 87, 6–10 (1990). 1688660 10.1073/pnas.87.1.6 PMC53188

[b34] Ng A. J., Mutsaers A. J., Baker E. K. & Walkley C. R. Genetically engineered mouse models and human osteosarcoma. Clin Sarcoma Res 2, 19 (2012). 23036272 10.1186/2045-3329-2-19 PMC3523007

[b35] Dahlin D. C. Bone Tumors: some aspects of their diagnosis. Am J Clin Pathol 25, 935–6 (1955). 14398658 10.1093/ajcp/25.8.935

[b36] Wagner E. R. . Defective osteogenic differentiation in the development of osteosarcoma. Sarcoma 2011, 325238 (2011). 21437219 10.1155/2011/325238 PMC3061279

[b37] Shimizu N. . Selective enhancing effect of early mitotic inhibitor 1 (Emi1) depletion on the sensitivity of doxorubicin or X-ray treatment in human cancer cells. J Biol Chem 288, 17238–52 (2013). 23645673 10.1074/jbc.M112.446351 PMC3682528

