# Supplementary material for: Ameloblastin induces tumor suppressive phenotype and enhances chemosensitivity to doxorubicin via Src-Stat3 inactivation in osteosarcoma
Source: Sci Rep. 2017 Jan 5;7:40187. doi: 10.1038/srep40187 (PMC5214574; doi:10.1038/srep40187)
Supplement: Supplementary Information [file srep40187-s1.pdf]

Ameloblastin induces tumor suppressive phenotype and enhances chemosensitivity to doxorubicin via Src-Stat3 inactivation in osteosarcoma

Toshinori Ando[1†], Yasusei Kudo[1,6†], Shinji Iizuka[1], Takaaki Tsunematsu[1,6], Hanako Umehara[1], Madhu Shrestha[1], Toshihiro Matsuo[7], Tadahiko Kubo[2], Shouji Shimose[5], Koji Arihiro[3], Ikuko Ogawa[4], Mitsuo Ochi[2], Takashi Takata[1*]

[1]Department of Oral and Maxillofacial Pathobiology, [2]Department of Orthopaedic Surgery, Institute of Biomedical and Health Sciences, Hiroshima University, Hiroshima, Japan, [3]Anatomical Pathology, [4]Center of Oral Clinical Examination, Hiroshima University Hospital, Hiroshima, Japan, [5]Division of Orthopaedic Surgery, National Hospital Organization Kure Medical Center, Kure, Japan, [6]Department of Oral Molecular Pathology, Institute of Health Biosciences, The University of Tokushima Graduate School, Tokushima, Japan, [7]Department of Orthopedic Surgery, Aichi Medical University, Nagakute, Aichi, Japan.

†These authors contributed equally to this work.

*To whom correspondence should be addressed. Takashi Takata D.D.S., Ph.D., Department of Oral and Maxillofacial Pathobiology, Basic Life Science, Institute of Biomedical and

Health Sciences, Hiroshima University, 1-2-3 Kasumi, Minami-ku, Hiroshima, Japan 734-8553. Tel: +81-82-257-5634, E-mail: ttakata@hiroshima-u.ac.jp

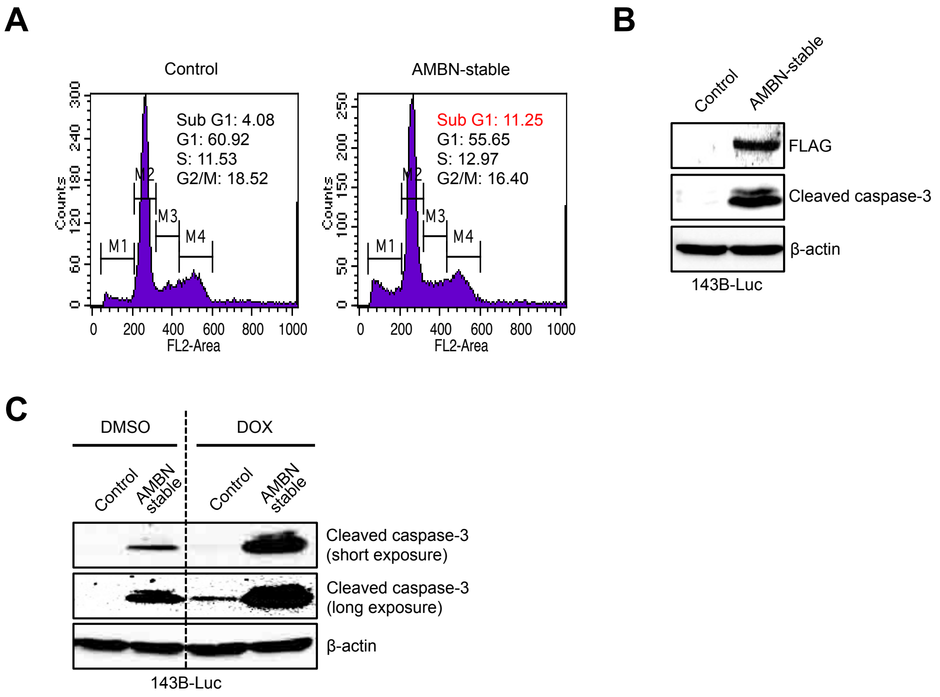

Figure S1. AMBN induces apoptosis and sensitivity to doxorubicin in osteosarcoma cells, related to Figure 1. **(A)** Representative results of cell cycle distributions analyzed by PI staining and FACS in control and AMBN-stable 143B-Luc cells are shown. **(B)** The expression of FLAG-AMBN and cleaved caspase-3 in control and AMBN-stable 143B-Luc cells was examined. **(C)** After the treatment with DMSO and doxorubicin (0.5 μg/mL) for 24 h in control and AMBN-stable 143B-Luc cells, cleaved caspase-3 expression was examined.

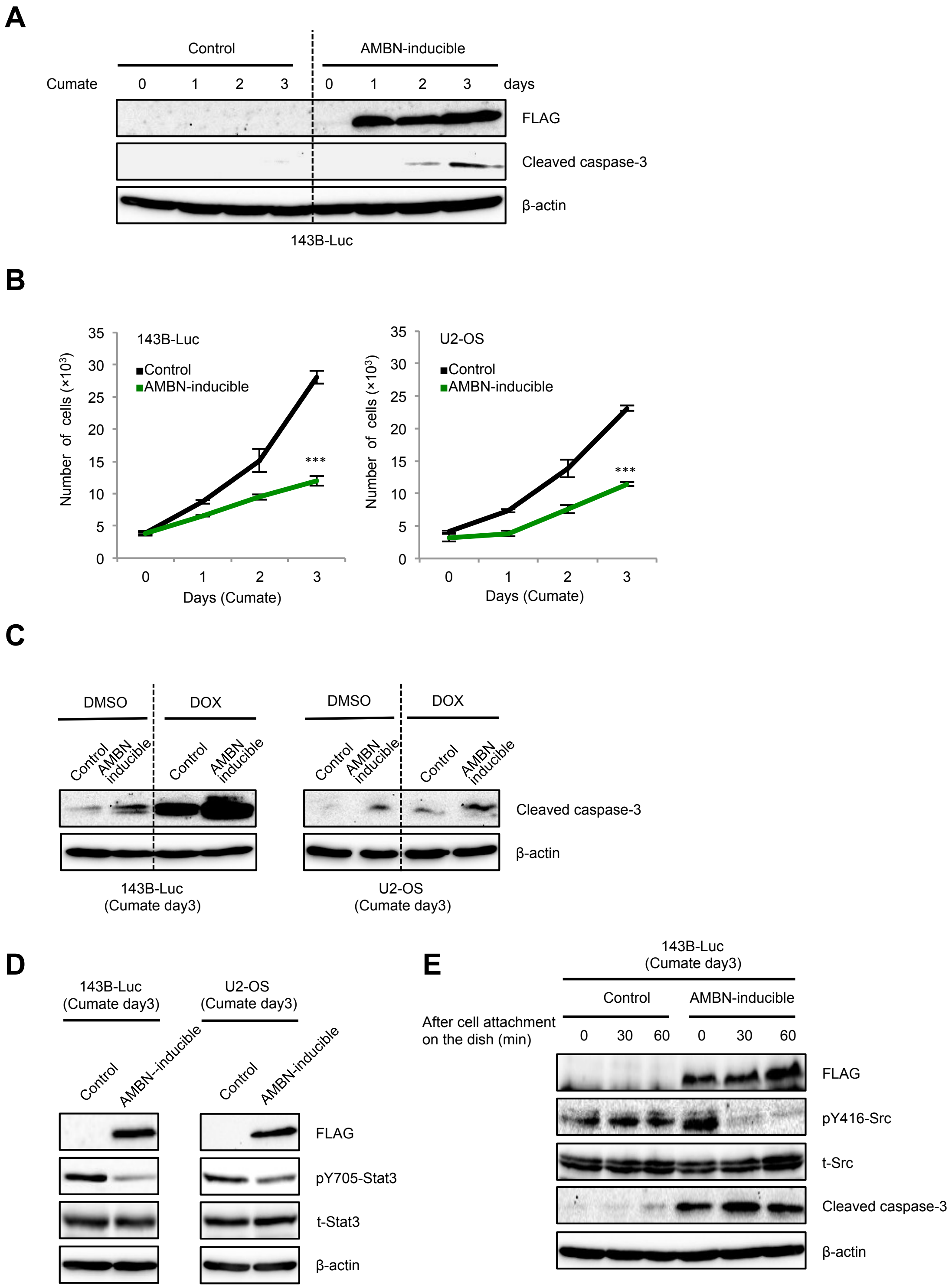
A
Control
AMBN-inducible
Cumate
0 1 2 3 0 1 2 3 days
FLAG
Cleaved caspase-3
β-actin
143B-Luc
B
143B-Luc
Control
AMBN-inducible
Number of cells (×10³)
Days (Cumate)
***
U2-OS
Control
AMBN-inducible
Number of cells (×10³)
Days (Cumate)
***
C
DMSO
DOX
Control
AMBN inducible
Cleaved caspase-3
β-actin
143B-Luc (Cumate day3)
U2-OS (Cumate day3)
D
143B-Luc (Cumate day3)
U2-OS (Cumate day3)
Control
AMBN-inducible
FLAG
pY705-Stat3
t-Stat3
β-actin
E
143B-Luc (Cumate day3)
Control
AMBN-inducible
After cell attachment on the dish (min)
0 30 60 0 30 60
FLAG
pY416-Src
t-Src
Cleaved caspase-3
β-actin

Figure S2. AMBN induces apoptosis and sensitivity to doxorubicin through the inactivation of Src-Stat3 pathway in osteosarcoma cells, related to Figure 1. **(A)** Control and AMBN-inducible 143B-Luc cells were cultured for 3 days with Cumate solution (300μg/mL). The cells were collected each day and the expression of FLAG-AMBN and cleaved caspase-3 was evaluated. **(B)** Control and AMBN-inducible 143B-Luc and U2-OS cells were cultured with Cumate solution, and cell growth was counted on days 0, 1, 2, and 3 (N=3). **(C)** Control and AMBN-inducible 143B-Luc and U2-OS cells were cultured with Cumate for 3 days, and these cells were treated with DMSO and doxorubicin (0.5 μg/mL) at last 24 h. The expression of FLAG-AMBN and cleaved caspase-3 was examined. **(D)** The expression of FLAG-AMBN, pY705-Stat3, total-Stat3 and cleaved caspase-3 was examined. **(E)** The expression of FLAG-AMBN, pY416-Src, total-Src and cleaved caspase-3 after attachment on the culture dish (0, 30, 60 minutes) was examined. Mean±SEM (**B**); ***, $P < 0.001$.

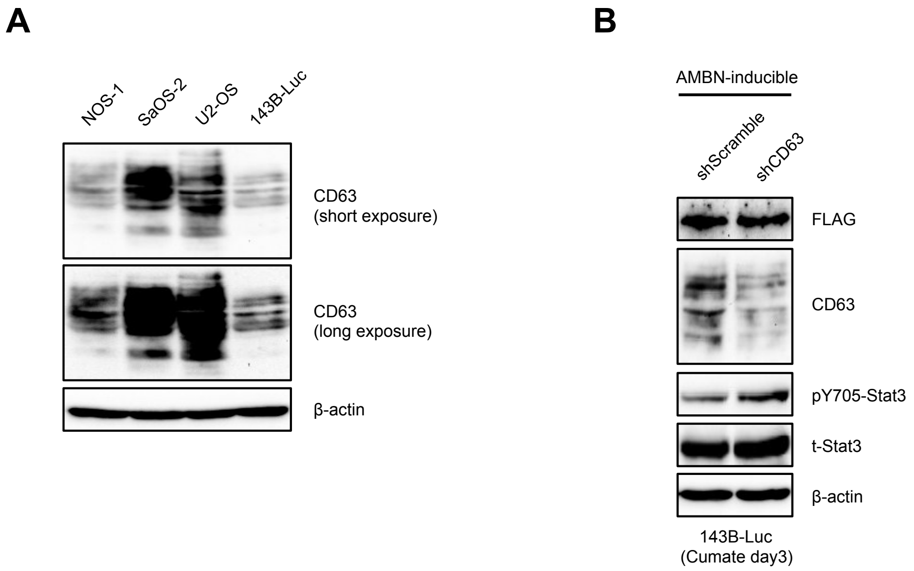

Figure S3. CD63 is expressed among human osteosarcoma cell lines and is needed for Stat3 inactivation induced by AMBN, related to Figure 1. **(A)** The expression of CD63 at the protein level in NOS-1, SaOS-2, U2-OS and 143-B Luc cells was examined. **(B)** shScramble and shCD63 were transfected into AMBN-inducible 143B-Luc cells. The expression of FLAG-AMBN, CD63, pY705-Stat3 and total-Src at the protein level in was examined.

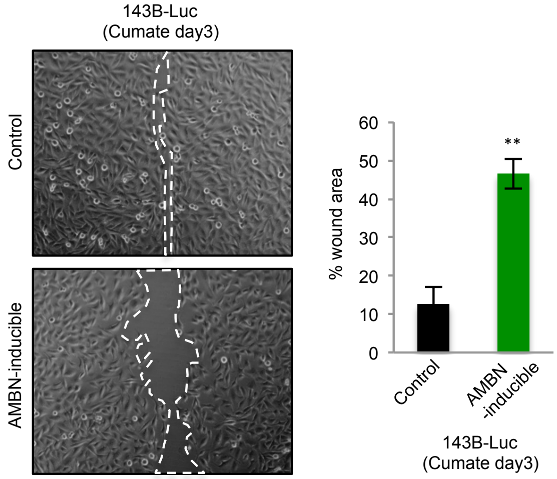

Figure S4. AMBN suppresses cell migration in osteosarcoma cells, related to Figure 2. Control and AMBN-inducible 143B-Luc cells were cultured with Cumate for 3 days. Cell migration activity was examined by wound healing assay. Representative images at 5 h are shown (left panels) and wound areas were quantified (right graph) (N=3). Original magnification of the left panels: ×100. Mean±SEM; **, $P < 0.01$.

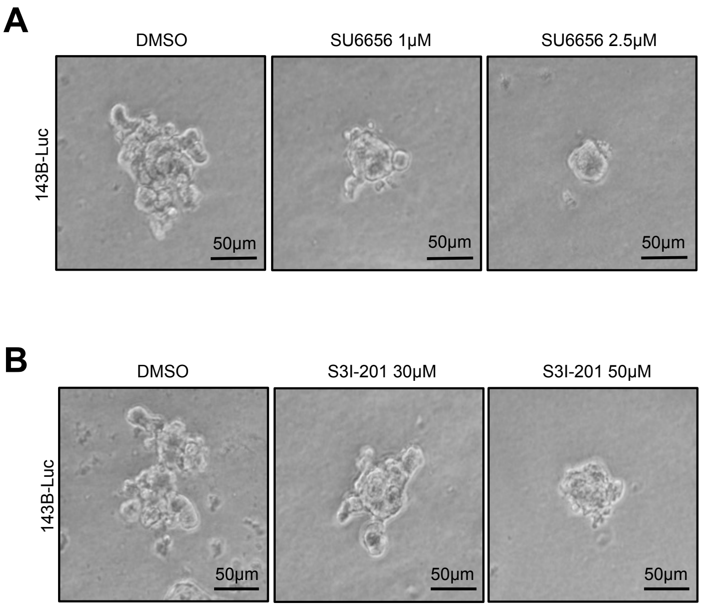

Figure S5. AMBN suppresses colony formation through the inactivation of Src-Stat3 pathway in osteosarcoma cells, related to Figure 3. **(A** and **B)** 143B-Luc cells were pretreated with SU6656 or S3I-201 at indicated concentrations for 24 h. Colony formation in pretreated 143B-Luc cells was analyzed and representative colonies were shown.

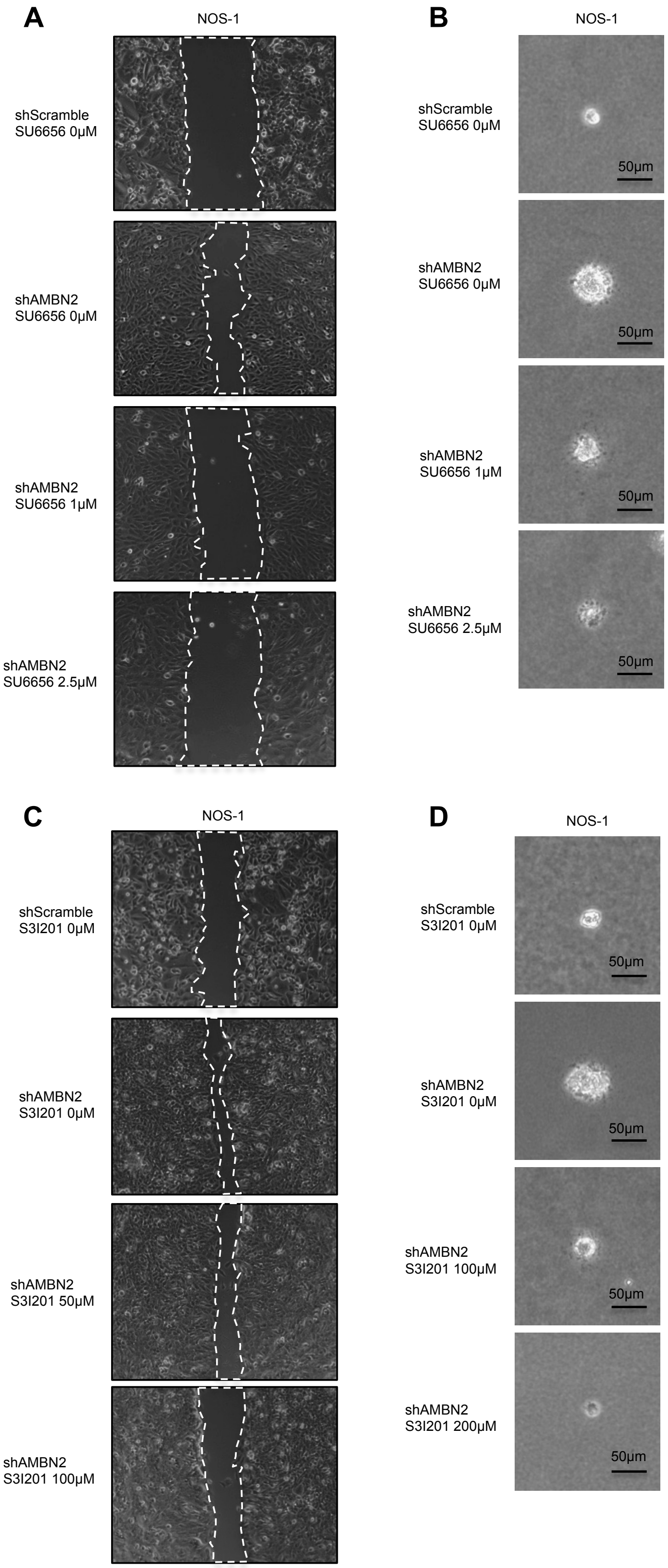
A
NOS-1
shScramble
SU6656 0μM
shAMBN2
SU6656 0μM
shAMBN2
SU6656 1μM
shAMBN2
SU6656 2.5μM
B
NOS-1
shScramble
SU6656 0μM
50μm
shAMBN2
SU6656 0μM
50μm
shAMBN2
SU6656 1μM
50μm
shAMBN2
SU6656 2.5μM
50μm
C
NOS-1
shScramble
S3I201 0μM
shAMBN2
S3I201 0μM
shAMBN2
S3I201 50μM
shAMBN2
S3I201 100μM
D
NOS-1
shScramble
S3I201 0μM
50μm
shAMBN2
S3I201 0μM
50μm
shAMBN2
S3I201 100μM
50μm
shAMBN2
S3I201 200μM
50μm

Figure S6. Knockdown of AMBN promotes cell migration and colony formation through Src-Stat3 axis in osteosarcoma cells, related to Figure 3. shAMBN-2 NOS-1 cells were pretreated with SU6656 at indicated concentrations. **(A** and **C)** Cell migration activity of pretreated NOS-1 cells was examined. Representative images of wound areas at 5 h are shown. **(B** and **D)** Colony formation in pretreated shAMBN-2 NOS-1 cells was analyzed and representative colonies are shown.

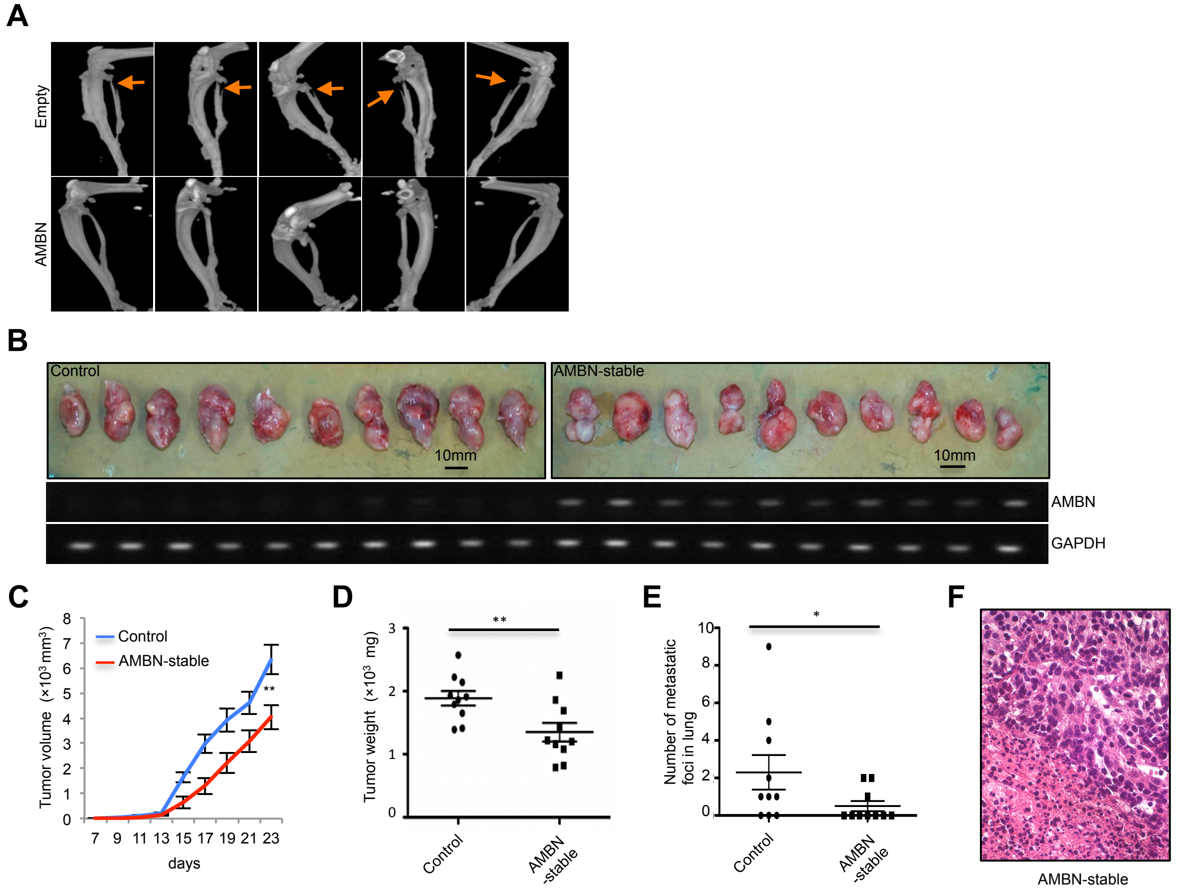

Figure S7. Stable AMBN overexpression inhibits tumor growth and pulmonary metastases *in vivo*, related to Figure 4. **(A)** Bone erosion of primary tumors in empty and AMBN group are shown. **(B)** The pictures of primary tumors in control and AMBN-stable group are shown (upper panel). AMBN mRNA in the primary tumor was evaluated (lower panel). **(C)** The volume of primary tumors at indicated days was measured (N=10). **(D)** The primary tumors were weighed after the sacrifice (N=10). **(E)** The number of metastatic foci in lung was counted by HE staining (N=10). **(F)** Representative necrotic area (lower left area: necrosis, right upper area: viable tumor cells) of primary tumor in AMBN-stable

group is shown. Original magnification: ×200. Mean±SEM (**C**-**E**); **, $P < 0.01$; *, $P < 0.05$.

Table S1. Clinical data and immunohistochemical results of AMBN in 37 osteosarcoma cases

Case No.	Age	Sex	Site	Subtype	Outcome	Follow up (months)	Lung metastasis	Ameloblastin expression
1	14	M	Femur	osteoblastic	DOD	11	-	+
2	20	M	Femur	fibroblastic	DOD	4	-	−
3	49	M	Femur	osteoblastic	CDF	89	-	−
4	16	M	Tibia	osteoblastic	CDF	82	-	+
5	14	F	Tibia	osteoblastic	DOD	36	+	−
6	13	M	Femur	osteoblastic	DOD	10	+	−
7	13	F	Femur	osteoblastic	DOD	5	-	−
8	19	M	Femur	teleangiectatic	CDF	145	-	−
9	15	F	Femur	osteoblastic	CDF	181	-	+
10	19	F	Femur	osteoblastic	DOD	13	+	−
11	14	M	Femur	small cell	CDF	90	-	−
12	13	M	Tibia	osteoblastic	NED	87	+	−
13	17	F	Femur	osteoblastic	CDF	69	-	−
14	42	M	Tibia	osteoblastic	DOD	43	-	−
15	28	F	Tibia	osteoblastic	CDF	156	-	+
16	24	M	Femur	osteoblastic	DOD	19	-	+
17	12	F	Pelvis	small cell	CDF	147	-	+
18	13	M	Femur	osteoblastic	NED	103	+	−
19	14	F	Femur	fibroblastic	CDF	55	-	+
20	10	M	Femur	osteoblastic	DOD	10	-	−
21	12	F	Femur	osteoblastic	CDF	124	-	+
22	15	M	Humerus	fibroblastic	DOD	37	-	−
23	14	M	Tibia	fibroblastic	CDF	109	-	+
24	69	M	Femur	parosteal os	CDF	73	-	−
25	16	F	Humerus	telangiectatic	CDF	102	-	−
26	15	M	Femur	osteoblastic	CDF	91	-	−
27	19	F	Tibia	osteoblastic	NED	97	+	+
28	12	M	Femur	osteoblastic	DOD	36	-	+
29	18	F	Tibia	osteoblastic	DOD	26	-	−
30	29	F	Femur	osteoblastic	CDF	55	-	−
31	15	F	Femur	osteoblastic	CDF	51	-	+
32	17	F	Tibia	fibroblastic	CDF	45	-	+
33	15	F	Femur	osteoblastic	CDF	46	-	+
34	88	F	Femur	fibroblastic	CDF	101	-	+
35	8	F	Femur	osteoblastic	DOD	-	+	−
36	13	F	Humerus	osteoblastic	AWD	61	+	−
37	14	M	Femur	osteoblastic	DOD	5	-	+

CDF: continuous disease-free, AWD: alive with disease, NED: no evidence of disease, DOD: dead of disease
